# FAM13A polymorphisms are associated with a specific susceptibility to clinical progression of oral cancer in alcohol drinkers

**DOI:** 10.1186/s12885-023-11052-5

**Published:** 2023-06-30

**Authors:** Ming-Ju Hsieh, Yu-Sheng Lo, Yun-Jung Tsai, Hsin-Yu Ho, Chia-Chieh Lin, Yi-Ching Chuang, Shu-Hui Lin, Mu-Kuan Chen

**Affiliations:** 1 Oral Cancer Research Center, Changhua Christian Hospital, Changhua, 500 Taiwan; 2 Program in Tissue Engineering and Regenerative Medicine, College of Medicine, National Chung Hsing University, Taichung, 402 Taiwan; 3 Graduate Institute of Biomedical Sciences, China Medical University, Taichung, 404 Taiwan; 4 Department of Surgical Pathology, Changhua Christian Hospital, Changhua, 500 Taiwan; 5 Translational Pathology Core Laboratory, Changhua Christian Hospital, Changhua, 500 Taiwan; 6 Department of Medical Laboratory Science and Biotechnology, Central Taiwan University of Science and Technology, Taichung, 406 Taiwan; 7 Department of Otorhinolaryngology, Head and Neck Surgery, Changhua Christian Hospital, No.135, Nanxiao St., Changhua City, Changhua County 500 Taiwan; 8 Department of Post-Baccalaureate Medicine, College of Medicine, National Chung Hsing University, Taichung, 402 Taiwan

**Keywords:** Polymorphism, Oral, FAM13A, Alcohol drinkers

## Abstract

**Background:**

Single nucleotide polymorphism (SNP) is a genetic variation that occurs when a single nucleotide base in the DNA sequence varies between individuals and is present in at least 1% of the population. Genetic variants in FAM13A are associated with different types of chronic respiratory diseases, including chronic obstructive pulmonary disease (COPD), cystic fibrosis (CF), and lung cancer. However, there is little literature on the association of FAM13A genotypes with oral cancer. Therefore, this project will explore the correlation between the FAM13A genotype and the formation of oral cancer.

**Methods:**

In this project, we will examine the presence of gene polymorphisms gene polymorphisms of rs1059122, rs3017895, rs3756050, and rs7657817 in the FAM13A gene exon, and combine the expression of these genes to try to clarify the impact of the FAM13A gene polymorphism on oral cancer. First, four loci (rs1059122, rs3017895, rs3756050, and rs7657817) of the FAM13A SNP were genotyped using TaqMan allelic discrimination.

**Results:**

By estimating OR and AOR, FAM13A exhibited different genotypic variables in four SNPs that were not statistically significant between controls and patients with oral cancer. The results of the general analysis showed that different distributions of allelic types did not affect clinical stage, tumour size, lymph node invasion, distant metastasis, and pathological differentiation status. However, in the alcohol drinking group specifically, patients with the rs3017895 SNP G genotype had a 3.17-fold (95% CI, 1.102–9.116; *p* = 0.032) increase in the well differentiated state of cells compared to patients with the A allele.

**Conclusions:**

Our results suggested that the SNP rs3017895 FAM13A could contribute to oral cancer. More sample studies are needed in the future to confirm our results and more functional studies are needed to investigate their relevant roles in the development of oral cancer.

## Introduction

The 11th most malignant cancer in the world is oral cancer, which has affected patients' health for decades [1]. The most common histological type of oral cancer, accounting for more than 90% of cases, is oral squamous cell carcinoma (OSCC) [2]. The prognosis for OSCC is suboptimal due to the high incidence of recurrence and metastasis, with an average 5-year survival rate of approximately 50% after treatment strategies such as surgery, radiation therapy, and chemotherapy [3, 4]. The development of OSCC is associated with poor survival due to genetic and environmental risk factors, including chewing betel quid (BQ), smoking, and alcohol consumption [5]. However, genetic and environmental risk factors have a synergistic effect on the incidence of OSCC [6]. Numerous genetic variants, such as single nucleotide polymorphisms (SNPs), are associated with various types of cancer, and gene polymorphisms have been found to contribute to the complexity of genetic regulatory changes that contribute to SNP-related cancer susceptibility [7]. The strength of SNP arrays in identifying key genetic abnormalities in cancer could provide a method to reliably segment tumours based on shared genetic abnormalities, to obtain the most appropriate treatment for patients [8].

FAM13A (family with sequence similarity 13, member A) is also known to be located on chromosome 4q22 and plays a role in various cellular processes such as cytoskeletal organisation, cell migration, and signal transduction [9]. Previous studies clarified that the highest expression of the FAM13A gene was detected in the brain and ovary, followed by the presence of the FAM13A gene in the lung and kidney [10]. The protein encoded by this gene has two functions of the coiled-coil domain and the presence of three specific nuclear localisation signals [11]. Genome-wide association studies (GWAS) have identified several variants of FAM13A genes that are strongly associated with different types of chronic respiratory diseases, including chronic obstructive lung disease (COPD), pulmonary fibrosis (PF), asthma, and lung cancer [12–15]. Previous studies have illustrated that SNPs in the 3’untranslated region (UTR) and FAM13A exons are associated with an increased risk of lung squamous cell carcinoma (LUSQ) [14, 16]. Furthermore, the expression of FAM13A increased significantly in cirrhotic tissue cells, and analysis showed that the G-A haplotype of the gene rs3017895-rs1059122 contributed significantly to the risk of liver cirrhosis [17]. In particular, the association of FAM13A rs1059122 with a reduced risk of breast cancer in a recessive model may contribute to susceptibility to breast cancer in the Chinese Han population [18]. However, the relationship between oral cancer and SNPs is not well understood. Our study identified the FAM13A gene as a haplotype of four SNPs (rs1059122, rs3017895, rs3756050 and rs7657817) with 3' untranslated region (UTR) and exons. Our study investigates the relationship between SNPs and OSCC in Asian populations.

## Materials and methods

### Patients and samples

This study collected data on 290 oral cancer cases and 290 cancer-free cases over an eight-year period from 2013 to 2021. The study was awarded to the Institutional Review Board of Changhua Christian Hospital (CCH) and Changhua Christian Hospital Biobank (IRB No. 200211), indicating that ethical considerations were considered. The study also noted that clinical staging, lymph node metastasis, and tumour cell differentiation in oral squamous cell carcinoma (OSCC) were explained using the standard TNM staging system of the American Joint Committee on Cancer (AJCC). The TNM system is a widely accepted method to describe the extent of cancer in a patient and is used by physicians to guide treatment decisions and predict patient outcomes. It involves assessing the size and extent of the primary tumour (T), the involvement of nearby lymph nodes (N), and the presence of distant metastases (M). By using the system, the study can more accurately classify and compare oral cancer cases with cancer-free cases [19]. The definition of chewing and drinking classifies people according to whether they chew betel nuts or drink alcohol. In this case, people who chewed betel quid or drank alcohol would be classified as having positive binary outcomes for these behaviours, not necessarily at the time of the study or survey. Definitions classify individuals according to whether they have smoked at least one cigarette per day in the past 3 months, indicating that they have a smoking habit.

### Genomic DNA extraction

Whole blood samples were collected during the hospitalisation of the patient and placed in sterile tubes containing EDTA. The collected samples were immediately centrifuged and stored at -80 °C. Genomic DNA was extracted from peripheral blood leukocytes using previously published methods using the QIAamp DNA Blood Mini Kit (Qiagen, Valencia, CA, USA) [20]. The extracts were dissolved in TE buffer (containing 10 mM trisaminomethane and 1 mM ethylenediaminetetraacetic acid; pH 7.8) and finally quantified by measuring optical density at a wavelength of 260 nm and stored at -20.

### Real-time PCR

Real-time PCR, also known as quantitative PCR (qPCR), uses fluorescent probes to monitor the amplification of the target sequence in real time during the PCR reaction. When the probe binds to the target sequence, the polymerase cleaves the probe, which converts the reporter molecule to the quencher. The reagent molecules are separated, resulting in fluorescence. The quantitative fluorescent PCR instrument detects the fluorescence intensity in each cycle of the PCR reaction, to achieve the quantification of the target nucleic acid [21]. Three FAM13A gene polymorphisms (rs3017895, rs3756050, and rs7657817) were detected in a previous study with Genesky's proprietary improved Multiligase Detection Reaction (iMLDR) [14]. Furthermore, the polymorphism of the FAM13A gene rs1059122 has been significantly reported in cirrhosis risk disease [17]. However, the role of FAM13A polymorphisms in oral cancer is unclear. The study systematically selected four SNPs from the FAM13A gene polymorphism (rs1059122, rs3017895, rs3756050 and rs7657817), which were analysed using quantitative real-time PCR with the ABI StepOne real-time PCR System (Applied Biosystems, Foster City, CA, USA), and the data was analysed using StepOne software v2.3. The final volume of each reaction was 5 μL and the contents were 2.5 μL TaqMan Genotyping Master Mix, 0.125 μL TaqMan probe mix, and 30 ng of genomic DNA. Real-time PCR experiments were setup with an initial denaturation step at 95° C for 10 min, followed by 40 cycles of amplification at 95° C for 15 s and 60° C for 1 min. Polymorphisms of the FAM13A gene were assessed by PCR as previously described [22].

### Bioinformatics analysis

This study aimed to analyse the association between the expression of the FAM13A gene and the clinical characteristics of head and neck squamous cell carcinoma (HNSCC) by downloading data from the Cancer Genome Atlas (TCGA) database through the University of California Santa Cruz (UCSC) Xena Functional Genomics Explorer, a web-based tool to explore and visualise genomic data. It is worth noting that users of the UCSC Xena Browser website ( https://xenabrowser.net/) [23] can access and analyse various genomic data sets through it. The article uses a browser search and retrieval of relevant TCGA data to analyse FAM13A gene expression and clinical features in HNSCC.

### Statistical analysis

As in the previous study [24], the collected data were analysed for clinical characteristics using IBM SPSS Statistics v22.0 (IBM, Armonk, NY, USA). We used Mann-Whitney U validation to assess demographically significant differences between OSCC cases and noncancer controls, and further analysed the variation of FAM13A levels in TCGA's HNSCC dataset. Furthermore, logistic regression was used to determine the odds ratio (OR) distribution of the FAM13A SNP distribution in OSCC cases versus noncancer controls. Multiple regression was used to calculate adjusted odds ratios (AOR) with 95% confidence intervals (CI) for the FAM13A SNP distribution, while logistic regression was used to evaluate the SNP after adjustment for chewing, smoking, and alcohol consumption of betel quid. A significance level of *p* < 0.05 was used to determine statistical significance.

## Results

### Characteristics of the cohort

This study explores the collection and analysis of 290 OSCC patients and 290 cancer-free controls. The study found that there were no significant differences in age distribution between the control group (people without oral cancer) and OSCC patients (*p* = 0.0751). As for the gender distribution, the percentage showed significant difference between control group and the OSCC patients (*p* < 0.0001). It might due to the incidence rate of male is much higher than female [25]. However, the study found that chewing, smoking, and drinking were significantly different between the two groups. This suggests that these lifestyle habits may be associated with an increased risk of developing oral cancer. Based on the eighth edition of the AJCC and TNM staging scheme [26], the patients had primary tumour predistribution (68.3%), lymph node metastases (76.2%) and no distant organ metastases (93.8%). Furthermore, Table 1 shows that around 83.1% of OSCC cases had moderate to poor cellular differentiation.

**Table 1:** The distributions of demographical characteristics and clinical parameters in 290 controls and 290 cases with OSCC

**Variable**	**Control (*N* = 290)**	**Patients (*N* = 290)**	***p* Value**
**Age (yrs.)** Mean ± SD	53.73 ± 7.75	55.08 ± 10.27	*p* = 0.0751
**Gender**			
Male	175 (60.3%)	280 (96.6%)	*p* < 0.0001*
Female	115 (39.7%)	10 (3.4%)	
**Betel nut chewing**			
No	279 (96.2%)	53 (18.3%)	*p* < 0.0001*
Yes	11 (3.8%)	237 (81.7%)	
**Cigarette smoking**			
No	268 (92.4%)	35 (12.1%)	*p* < 0.0001*
Yes	22 (7.6%)	255 (87.9%)	
**Alcohol drinking**			
No	283 (97.6%)	142 (49.0%)	*p* < 0.0001*
Yes	7 (2.4%)	148 (51.0%)	
**Stage**			
I + II		173 (59.7%)	
III + IV		117 (40.3%)	
**Tumor T status**			
T1 + T2		198 (68.3%)	
T3 + T4		92 (31.7%)	
**Lymph node status**			
N0		221 (76.2%)	
N1 + N2 + N3		69 (23.8%)	
**Metastasis**			
M0		272 (93.8%)	
M1		18 (6.2%)	
**Cell differentiation**			
Well differentiated		49 (16.9%)	
Moderately or poorly differentiated		241 (83.1%)	

*N* Number. Mann–Whitney U test was used between OSCC patients and non-cancerous controls

^*^p value < 0.05 as statistically significant

### The polymorphism of the FAM13A gene can affect the occurrence and progression mechanism

To explore the distribution of FAM13A genotypes, four SNPs (rs1059122, rs3017895, rs3756050, and rs7657817) and clinical symptoms were separately analysed. The statistical analysis used was the estimation of the OR and its 95% CI using a logistic regression model. Additionally, we use personal habits (drinking, chewing nut beets, and smoking) as AOR as a secondary analysis target and incorporate different variables into the calculation results. We revealed, by estimating the OR and AOR, that FAM13A exhibited different genotypic variables in four SNPs that were not statistically significant between controls and patients with oral cancer, as shown in Table 2. We then further explored whether there is an association between different variants of SNP rs3017895 and specific characteristics of patients with OSCC, such as tumour size or lymph node involvement. By examining these relationships, we aim to determine whether the SNP could be used as a marker to predict the development and progression of OSCC. The results of this analysis would have been presented in Table 3, which likely lists the various categories of clinical and pathological characteristics that were studied. The study found that different variants (allele types) of SNP rs3017895 were not significantly associated with various clinicopathological characteristics such as clinical stage, tumour size, lymph node invasion, distant metastasis, metastasis, and pathological differentiation status of patients with OSCC. This means that the distribution of different types does not appear to have any effect on the development or progression of OSCC or tumour characteristics in patients with OSCC. The lack of a significant association suggests that this particular SNP may not be a useful marker to predict the severity of OSCC or the clinical outcome. To further explore whether drinking alcohol, chewing betel nuts, and smoking are considered risk factors for SNP rs3017895 and OSCC, we calculated the correlation between patients with different genotypes. Our study indicated that subjects who chewed betel quid and smoked were not associated with clinical stage, tumour size, lymph node metastasis, distant metastasis, or cell differentiation status FAM13A SNP subgroup rs3017895. Especially in the alcohol drinking group, patients with the SNP G genotype were found to have a 3.17-fold increase in the highly differentiated state of cells (95% CI, 1.102-9.116; *p* = 0.032) and a significantly lower incidence of distant metastases (95% CI, 0.056-0.876; *p* = 0.032) compared to patients with the A allele, as shown in Table 4. The above results suggest that the characteristics of patients with different habits are associated with FAM13A SNP and environmental risk and oral cancer progression.

**Table 2:** Distribution of genotype frequencies in FAM13A SNPs in cases of OSCC group

**Variable**	**Control (*N* = 290)**	**Patients (*N* = 290)**	**OR**^**a**^** (95% CI)**	**AOR**^**b**^** (95% CI)**
**rs1059122**				
AA	79 (27.2%)	92 (31.7%)	1.000	1.000
AT	149 (51.4%)	151 (52.1%)	0.870 (0.597–1.268)	0.902 (0.445–1.825)
TT	62 (21.4%)	47 (16.2%)	0.651 (0.401–1.056)	0.702 (0.275–1.793)
AT + TT	211 (72.8%)	198 (68.3%)	0.806 (0.563–1.152)	0.848 (0.432–1.664)
**rs3017895**				
AA	146 (50.3%)	125 (43.1%)	1.000	1.000
AG	126 (43.4%)	139 (47.9%)	1.289 (0.918–1.809)	1.256 (0.654–2.412)
GG	18 (6.2%)	26 (9.0%)	1.687 (0.884–3.221)	1.333 (0.384–4.625)
AG + GG	144 (49.7%)	165 (56.9%)	1.338 (0.965–1.856)	1.267 (0.675–2.377)
**rs3756050**				
TT	77 (26.6%)	95 (32.8%)	1.000	1.000
TC	157 (54.1%)	147 (50.7%)	0.695 (0.426–1.133)	0.877 (0.339–2.269)
CC	56 (19.3%)	48 (16.6%)	0.759 (0.521–1.105)	1.095 (0.538–2.230)
TC + CC	213 (73.4%)	195 (67.2%)	0.742 (0.519–1.061)	1.039 (0.526–2.053)
**rs7657817**				
CC	159 (54.8%)	172 (59.3%)	1.000	1.000
CT	121 (41.7%)	96 (33.1%)	0.733 (0.520–1.034)	0.777 (0.405–1.491)
TT	10 (3.4%)	22 (7.6%)	2.034 (0.934–4.428)	1.607 (0.396–6.517)
CT + TT	131 (45.2%)	118 (40.7%)	0.833 (0.599–1.157)	0.850 (0.456–1.586)

*N* Number. Logistic regression models were used to estimate odds ratios (OR) and their corresponding 95% confidence intervals. ^b^ Multiple logistic regression models were used to estimate adjusted odds ratios (AOR) and their corresponding 95% confidence intervals. These models were used after controlling for the potential confounding effects of betelnut chewing, alcohol consumption, and tobacco consumption. The adjusted odds ratio provides a more accurate estimate of the true association between the variables of interest by taking into account the effects of other factors that may influence the outcome

**Table 3:** Clinical status and FAM13A rs3017895 genotype frequencies in cases of the OSCC group

**Variable**	**FAM13A (rs3017895)**			
	**AA (%)** **(*N* = 125)**	**AG + GG (%)** **(*N* = 165)**	**OR**^**a**^** (95% CI)**	***p* Value**
**Clinical stage**				
Stage I/II	71 (56.8%)	102 (61.8%)	1.000	*p* = 0.389
Stage III/IV	54 (43.2%)	63 (38.2%)	0.812 (0.506–1.303)	
**Tumor size**				
T1 + T2	82 (65.6%)	116 (70.3%)	1.000	*p* = 0.394
T3 + T4	43 (34.4%)	49 (29.7%)	0.806 (0.490–1.325)	
**Lymph node metastasis**				
No	94 (75.2%)	127 (77.0%)	1.000	*p* = 0.726
Yes	31 (24.8%)	38 (23.0%)	0.907 (0.527–1.563)	
**Distant metastasis**				
No	115 (92.0%)	157 (95.2%)	1.000	*p* = 0.275
Yes	10 (8.0%)	8 (4.8%)	0.586 (0.224–1.531)	
**Cell differentiation**				
Well	22 (17.6%)	27 (16.4%)	1.000	*p* = 0.781
Moderate/poor	103 (82.4%)	138 (83.6%)	1.092 (0.588–2.025)	

*N* Number. A logistic regression model was used to estimate odds ratios (OR) and their 95% confidence intervals

**Table 4:** Clinical statuses and frequencies of FAM13A rs3017895 genotypes in cases of the OSCC group among chewing betel nut, smoking and drinking alcohol

**Variable**	**FAM13A (rs3017895)**								
	**Betel nut chewing (*N* = 237)**			**Cigarette smoking (*N* = 255)**			**Alcohol drinking (*N* = 148)**		
	**AA (%)** **(*N* = 106)**	**AG + GG (%)** **(*N* = 131)**	***p* Value**	**AA (%)** **(*N* = 109)**	**AG + GG (%)** **(*N* = 146)**	***p* Value**	**AA (%)** **(*N* = 59)**	**AG + GG (%)** **(*N* = 89)**	***p* Value**
**Clinical stage**									
Stage I/II	58 (54.7%)	78 (59.5%)	*p* = 0.455	63 (57.8%)	92 (63.0%)	*p* = 0.399	31 (52.5%)	48 (53.9%)	*p* = 0.868
Stage III/IV	48 (45.3%)	53 (40.5%)		46 (42.2%)	54 (37.0%)		28 (47.5%)	41 (46.1%)	
**Tumor size**									
T1 + T2	67 (63.2%)	89 (67.9%)	*p* = 0.445	74 (67.9%)	105 (71.9%)	*p* = 0.487	38 (64.4%)	55 (61.8%)	*p* = 0.748
T3 + T4	39 (36.8%)	42 (32.1%)		35 (32.1%)	41 (28.1%)		21 (35.6%)	34 (38.2%)	
**Lymph node metastasis**									
No	79 (74.5%)	98 (74.8%)	*p* = 0.961	80 (73.4%)	115 (78.8%)	*p* = 0.318	43 (72.9%)	63 (70.8%)	*p* = 0.782
Yes	27 (25.5%)	33 (25.2%)		29 (26.6%)	31 (21.2%)		16 (27.1%)	26 (29.2%)	
**Distant metastasis**									
No	98 (92.5%)	126 (96.2%)	*p* = 0.218	99 (90.8%)	140 (95.9%)	*p* = 0.108	51 (86.4%)	86 (96.6%)	*p* = 0.032^*,a^
Yes	8 (7.5%)	5 (3.8%)		10 (9.2%)	6 (4.1%)		8 (13.6%)	3 (3.4%)	
**Cell differentiation**									
Well	21 (19.8%)	21 (16.0%)	*p* = 0.449	21 (19.3%)	24 (16.4%)	*p* = 0.558	11 (18.6%)	6 (6.7%)	*p* = 0.032*^,b^
Moderate/poor	85 (80.2%)	110 (84.0%)		88 (80.7%)	122 (83.6%)		48 (81.4%)	83 (93.3%)	

*N* Number

^*^p value < 0.05 as statistically significant

^a^OR (95% CI): 0.222 (0.056–0.876)

^b^OR (95% CI): 3.170 (1.102–9.116)

### Clinical and Functional Insights from FAM13A to OSCC

**Fig. 1:**
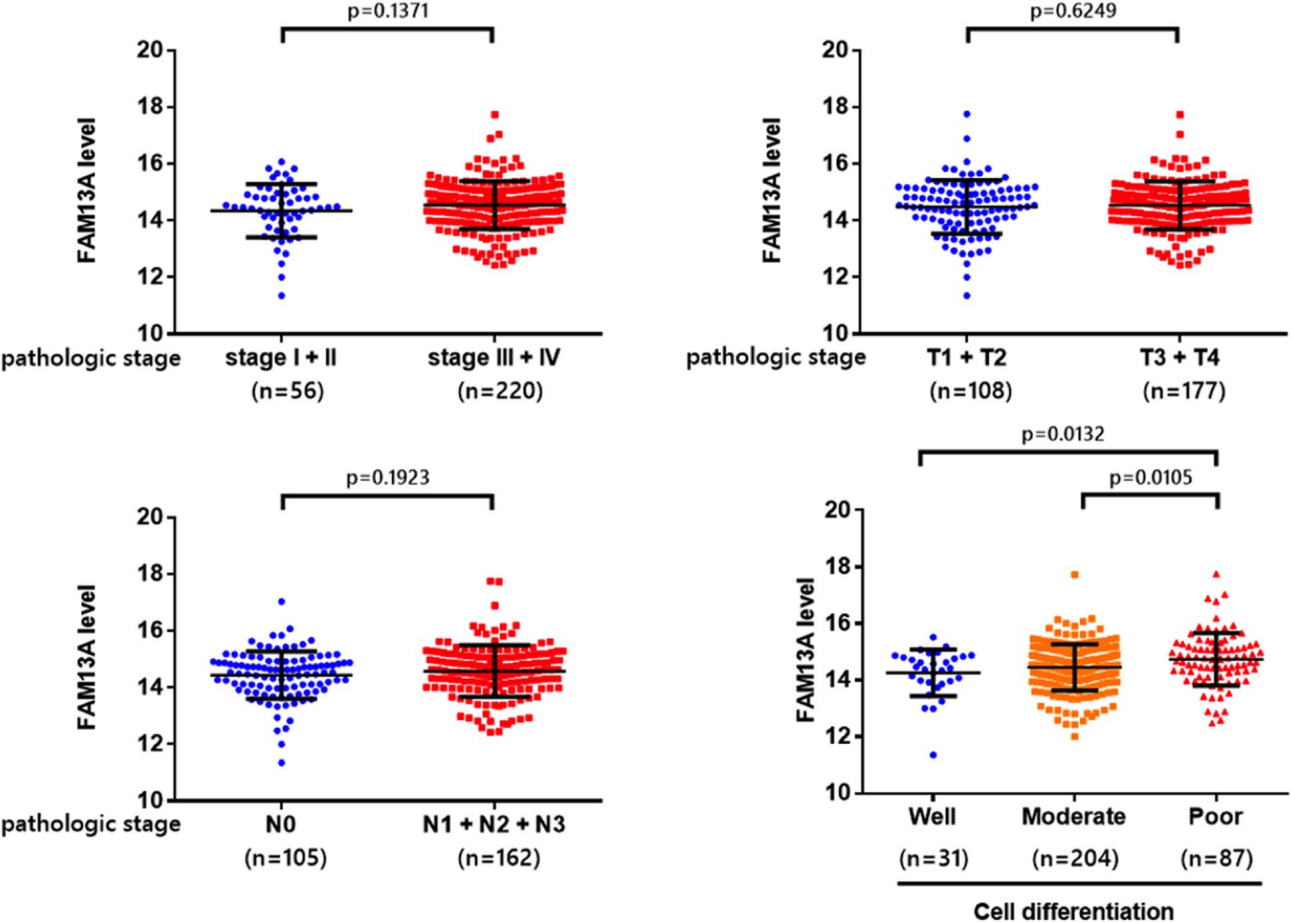
The purpose of the study was to explore the potential relationship between the expression level of the FAM13A gene and various clinicopathological parameters in cases of HNSCC. The relationship between the expression of the FAM13A gene and different aspects of HNSCC was investigated using data from the TCGA database. Specifically, the study examined the correlation between FAM13A expression and clinical stage (**A**), tumour size (**B**), lymph node metastasis (**C**), and cell differentiation (**D**) in HNSCC. Results with a *p*-value of less than 0.05 were considered statistically significant

Previous studies have shown a genetic association between FAM13A and oral cancer, and we explore the clinical features of this gene using the TCGA dataset, as shown in Fig. 1. We did not observe significant differences in the FAM13A gene between various clinical characteristics, including clinical stage (*p* = 0.1371), tumour (*p* = 0.6249), and lymph node metastasis (*p* = 0.1923). However, we did find that the cell differentiation status was significantly different between the good and poor status (*p* = 0.0132), as well as between the intermediate and poor status (*p* = 0.0105). These findings suggest that the degree of cell differentiation may be a more important predictor of severity and clinical outcome than the expression of the FAM13A gene.

## Discussion

Humans have a very high degree of genetic similarity, with over 99% identity in their genome sequence. This means that differences between individuals are typically due to variations in small sections of their DNA, such as tandem repeats, insertion or deletion polymorphisms, and single nucleotide polymorphisms (SNPs). These variations account for less than 1% of the overall genetic material and contribute to the diversity of the human population [27]. GWAS have identified specific SNPs that are associated with the development of cancer and its various characteristics. Cancer Genome Atlas (TCGA) has also shown that there are differences in DNA sequence between tumour cells and normal cells [28, 29]. SNPs have a highly modulated susceptibility to disease by the interaction of human exposure to environmental factors and specific allelic variants. Several conclusions about gene-environment interactions illuminate their combined impact on human cancer incidence and/or prevalence [30]. Together, these findings suggest that genetic variations play an important role in cancer development and progression and may provide information on potential targets for diagnosis, treatment, and prevention.

Oral cancer is a common and serious health problem. Smoking and alcohol consumption are considered major factors in the development of oral cancer and are among the leading causes of death related to this disease [31]. As noted in previous studies, several studies have demonstrated familial clustering, suggesting a role for genetic components in the development of oral cancer [32]. High genetic influence can lead to the development of up to 10% of cancers. There has been much recent research evidence showing the association between oral cancer and SNPs in different genes. Exploring specific genetic polymorphisms of key genes related to oral carcinogenesis has been a major area of research. Polymorphisms in glutathione S-transferase (GST) genes (GSTM1, GSTT1, and GSTP1) and their interaction with environmental factors such as tobacco and alcohol influence susceptibility to HNSCC [33]. Singh et al. demonstrated that alcohol consumption resulted in a four-fold increase in risk in patients with GSTM1 null genotype compared to non-drinkers [34]. The CYP1A1 gene encodes an aromatic hydrocarbon hydroxylase that induces the biotransformation of aromatic tobacco carcinogens and may play a key role in the pathogenesis of oral squamous cell carcinoma (OSCC) through the MspI polymorphism. The MspI SNP in the CYP1A1 gene indicated a 34% increased risk of head and neck cancer in carriers of the TC and CC genotype compared to carriers of carriers of TT carriers [35]. The main enzymes involved in alcohol metabolism are alcohol dehydrogenase (ADH) and acetaldehyde dehydrogenase (ALDH), and noncoding variants of the ADH and ALDH genes can also affect alcohol metabolism [36]. It is becoming increasingly clear that null ALDH alleles lead to elevated acetaldehyde levels and are believed to increase the risk of head and neck cancer [37].

Previous studies have confirmed a relationship between FAM13A SNPs and various cancers, such as non-small cell lung cancer, renal cell carcinoma, cervical cancer, and breast cancer [12, 18, 38, 39]. The results of this study indicate a significant difference in the SNP located in the FAM13A gene between oral cancer patients and the control group. We first explored individual habit-adjusted odds ratios, which did not differ significantly between the control and OSCC patient groups. This result suggests that an individual's personal lifestyle habits and their genetic makeup, particularly the FAM13A variant rs3017895, may play an important role in the development of oral cancer. Our analysis is consistent with previous research that has shown a correlation between the minor G allele of the FAM13A variant rs3017895 and an increased susceptibility to lung cancer among the Han Chinese Han population [14]. Follow-up studies are needed with larger control and patient groups to confirm the importance of FAM13A SNP rs3017895 in oral cancer. Therefore, further analysis will be performed on the FAM13A variant rs3017895.

Numerous studies have shown that personal habits, such as drinking, smoking, and chewing betel nuts, are strongly associated with the development of oral cancer [40–42]. However, when comparing the effects of different alleles on personal habits, only alcohol consumption showed a statistically significant difference. Furthermore, the association between alcohol consumption and oral cancer is multifactorial and is influenced by various factors, including genetics, lifestyle habits, and environmental exposure. The enzymes involved in alcohol metabolism are mainly ADH and ALDH, and noncoding variants of ADH and ALDH genes may also affect alcohol metabolism. In particular, slow ethanol metabolism has been associated with an increased risk of head and neck cancer, particularly in people who slowly metabolise alcohol slowly [43]. Previous studies have shown that COPD genome-wide association studies have identified genetic risk variants in FAM13A [44]. Alcohol contributes to co-carcinogenesis or contributes to carcinogenesis, especially acetaldehyde, which has been shown to alter DNA-associated epigenetic alterations in head and neck cancer [45, 46]. Therefore, we investigated the relationship between the FAM13A rs3017895 polymorphism and personal habits, as well as clinical status, in patients with oral cancer. Especially in patients with alcohol consumption, the highly differentiated state of cells in patients with the G genotype increased 3.17 times (95% CI, 1.102–9.116) compared to patients with the SNP A genotype rs3017895. Consistent with the above studies, we demonstrate that FAM13A polymorphisms have strong effects and significant differences in the susceptibility of oral cancer to alcohol consumption. In general, our findings suggest that SNP FAM13A rs3017895 may be a key factor in predicting tumour recurrence, target therapy response, and drug toxicity in patients with oral cancer. More research is needed to better understand the correlation between this SNP and other common somatic genetic changes in oral cancer.

## Conclusions

Based on the experimental results, it has been confirmed that there is an association between the SNP rs3017895 located in the FAM13A gene and the development of OSCC, as well as poorer clinical stage in patients with OSCC. This suggests that this specific genetic variation may be a potential biomarker of OSCC and could be useful to identify people who may be at increased risk of developing the disease or to monitor the progression of the disease in patients with OSCC. Especially in the drinking group, it was found that patients with the SNP G genotype had a 3.17-fold increase in the state of highly differentiated cells and a significantly lower incidence of distant metastasis compared to patients with the A allele. Finally, the role of the FAM13A SNP provides evidence for further investigation of the utility of the genetic marker in diagnosis and prevention.

## Data Availability

The datasets generated during and analysed during the current study are available from the corresponding author on reasonable request.
